# Brain Activity at 70–80 Hz Changes during Olfactory Stimulation Protocols in *Drosophila*

**DOI:** 10.1371/journal.pone.0012867

**Published:** 2010-09-22

**Authors:** Lucia L. Prieto-Godino, Gonzalo G. de Polavieja

**Affiliations:** 1 Department of Theoretical Physics, Universidad Autónoma de Madrid, Madrid, Spain; 2 Instituto ‘Nicolás Cabrera’ de Física de Materiales, Universidad Autónoma de Madrid, Madrid, Spain; Center for Genomic Regulation, Spain

## Abstract

Oscillatory and synchronized activities in the mammalian brain have been correlated with the execution of complex cognitive tasks. Similar oscillations have been observed in local field potentials (LFPs) in flies, in this case correlated with different attentional states. To further test the significance of these oscillations we recorded LFPs from the brain of *Drosophila melanogaster* as it responded to the presentation of olfactory stimuli. We find that responses in the 70–80 Hz range increase during olfactory stimulation. Recurrent stimulation specifically decreased the power of LFPs in this frequency range. Delivery of electric shocks before olfactory stimulation modulated LFPs in the 70–80 Hz range by evoking a transient increase. These results suggest that these signals are a simple neuronal correlate of higher-order olfactory processing in flies.

## Introduction

Electroencephalogram and local field potential (LFP) oscillations generally indicate periodic coherent synchronization of neuronal assemblies [1]–[3]. Oscillations have been found in systems as disparate as mollusks [2], moths [4], locusts [5], [6], rats and mice [7], [8], suggesting a fundamental role in computations carried out during higher-order processing. Oscillatory and synchronized activities in the mammalian brain have been correlated with distinct behavioural states or the execution of complex cognitive tasks, and are proposed to participate in the ‘binding’ of individual features into more complex percepts [3], [9]–[11]. Similar oscillations have been observed in LFP recordings from the first and second relay centers for olfactory information in insects. The nature and function of these oscillations have been investigated in more detail in bees, locusts, moths and flies [4], [6], [12], [13]. These studies show that oscillations caused by synchronized activity of projection neurons (first interneurons of the insect olfactory pathway), and that GABAergic local interneurons in the antennal lobe are essential for such synchronization. In these systems synchrony is essential for fine odour recognition at the cellular [6] and behavioural levels [12]. Direct evidence for the role of these oscillations in neuronal processing comes from studies in the locust [6]. Specifically, disrupting the mushroom body oscillations by application of a GABA antagonist to the antennal lobe produces a loss of information in the third olfactory synapse without affecting the information content in the spike train of its presynaptic neurons. However it is not known whether and how the oscillations observed in insect sensory systems and the ones recorded from mammals during the execution of complex cognitive tasks are functionally and computationally related. A first step in this direction is to find out whether oscillations occur in higher brain structures of insects during the performance of complex cognitive tasks, involving processes such as selective attention [14], contextual generalization [15], or formation of ‘sameness’ and ‘difference’ concepts [16]. Recently two reports have correlated in flies LFP oscillatory activity recorded centrally in the brain with different behavioural states, as it happens in mammals [17], [18]. These studies found that conspicuousness of different visual objects modulates the oscillatory activity recorded from central brain structures in the 20–30 Hz range. The output of a subset of neurons in the mushroom body is required for both the oscillatory activity and the behavioural response.

Interestingly, it has also been reported an increase in the 70–80 Hz range in LFPs recorded in the medial protocerebrum (mpc) during olfactory stimulation [18]. Here we pursue further the study of this signal during different olfactory protocols, demonstrating that LFP oscillations recorded in the mpc are not exclusively modulated by visually guided behaviours. Our results suggest a more general role for mpc oscillations in cognitive tasks.

## Results

### Evoked responses

**Figure 1:**
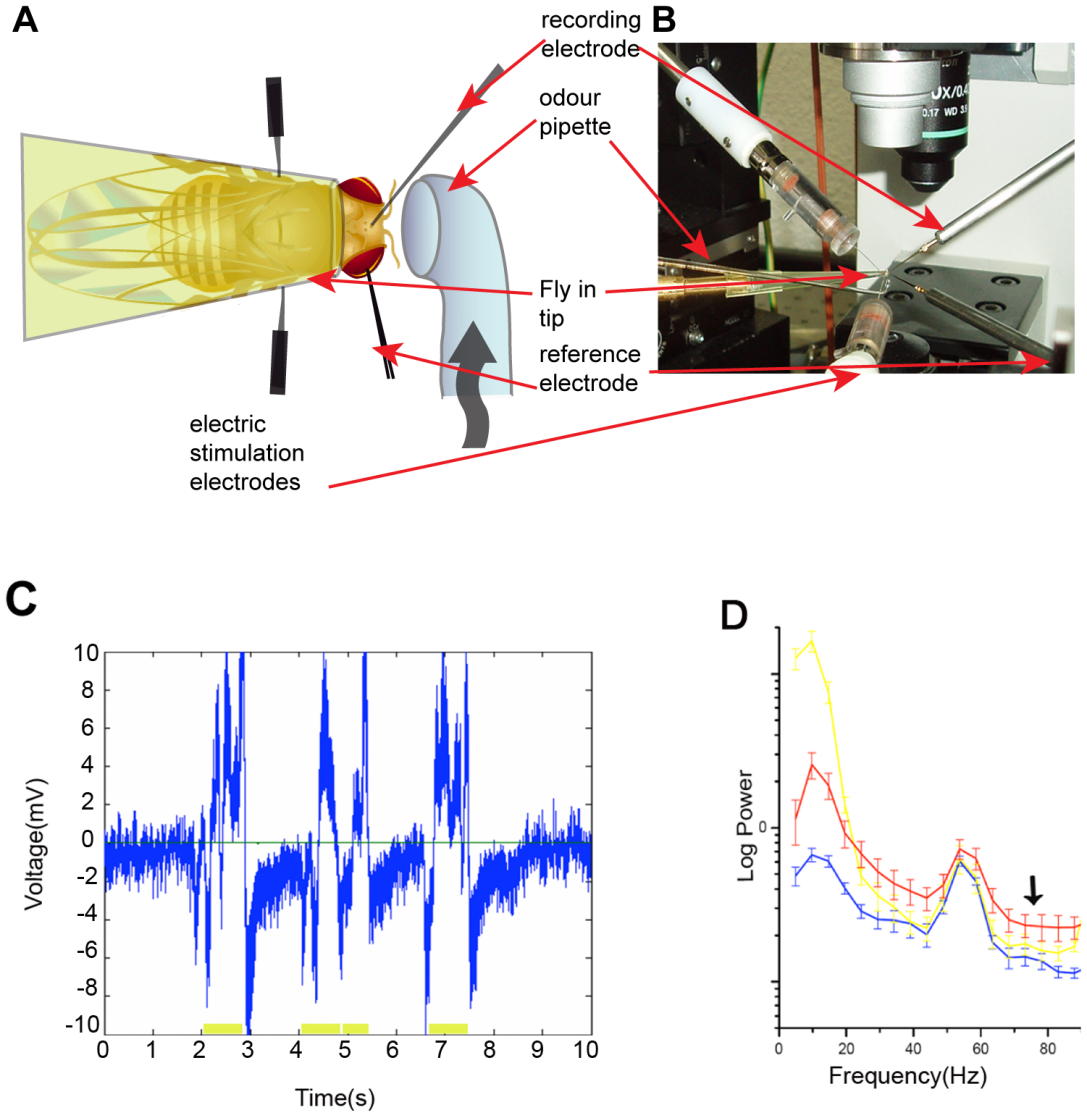
Experimental set-up. (A,B) Flies were placed in a plastic pipette tip and held in place with low melting temperature wax. Tungsten electrodes were implanted into the right retina and medial protocerebrum. Odour was delivered to the antenna with a bent glass pipette. Electrical shocks were delivered to the fly thorax with silver electrodes through openings in the plastic pipette filled with conductive gel. (C) A sample 10 s of raw signal (blue trace) while stimulating with a small flashlight. Large amplitude deflections in the potential are responses to light flashes (yellow bars at the bottom of the figure). (D) Sample power spectrum of brain frequencies (0–90 Hz) for non-stimulation condition (blue trace), compared to the spectrum during light (yellow trace), and odour (red trace) stimulation. The arrow points to the 70–80 Hz response to odours. The peak apparent at 55 Hz is due to environmental electrical noise and probably also to some physiological signals. Every trace represents the mean with its standard deviation calculated from several measurements on the same fly. Stimuli and non-stimulation intervals were presented randomly (Methods). Number of measurements for each condition: n = 7 (no stimulation, blue trace), n = 5 (light stimulation, yellow trace), n = 7 (odour stimulation, red trace).

Flies were placed in a pipette tip to restrict their movements and the antennae were left free and accessible for olfactory stimulation. Odours were applied through a glass micropipette directed at the antennae, and LFP responses to olfactory stimuli were recorded from their brains. Brain activity was recorded as a voltage differential between an electrode placed in the right compound eye and an electrode inserted into the medial protocerebrum (mpc) (**Fig. 1A and B**). To ascertain that the reference electrode was correctly placed, and to check that flies responded to external stimuli, flies were assayed for light responsiveness before every recording. As our reference electrode is placed in the eye, and the recording electrode is in the brain, flashes of light produce high amplitude, low frequency deflections in the recording. Only flies that responded to light stimuli were used for further experiments (**Fig. 1C**). A spectral analysis of the brain recordings, in the absence of stimuli and during light and olfactory stimulation, revealed increased power across all frequencies (1–90 Hz; **Fig. 1D**) in the stimulated conditions compared to brain activity at rest. As reported before [14], [18] there was an increased power at low frequencies (below 10 Hz) when a visual stimulus was presented. This signal is thought to come primarily from the optic lobe [18], and has been associated with the optomotor response, but as the strength of the signal varies in short term memory mutants, probably some central processing is involved in its regulation [14]. We also found an increased power at medium frequencies (10–20Hz), and at high frequencies (70–80 Hz), when an odour was presented (**Fig. 1D**). An increase in power at medium frequencies (10–20 Hz) has also been reported in LFP recordings of the antennal lobe and mushroom body during olfactory stimulation in several insect species, including *Drosophila*, and it is thought to be caused by coherent firing of projection neurons mediated by local interneurons inhibition [4], [5], [12], [13]. Here we pursued the study of the high frequency signal (70–80 Hz). Due to the method and sampling frequency used, the Fourier transform yields two values in the 70–80 Hz range at 73 and 78 Hz, the average of which is defined as the 70–80 Hz power.

### Odour stimulation modifies brain activity at 70–80 Hz

**Figure 2:**
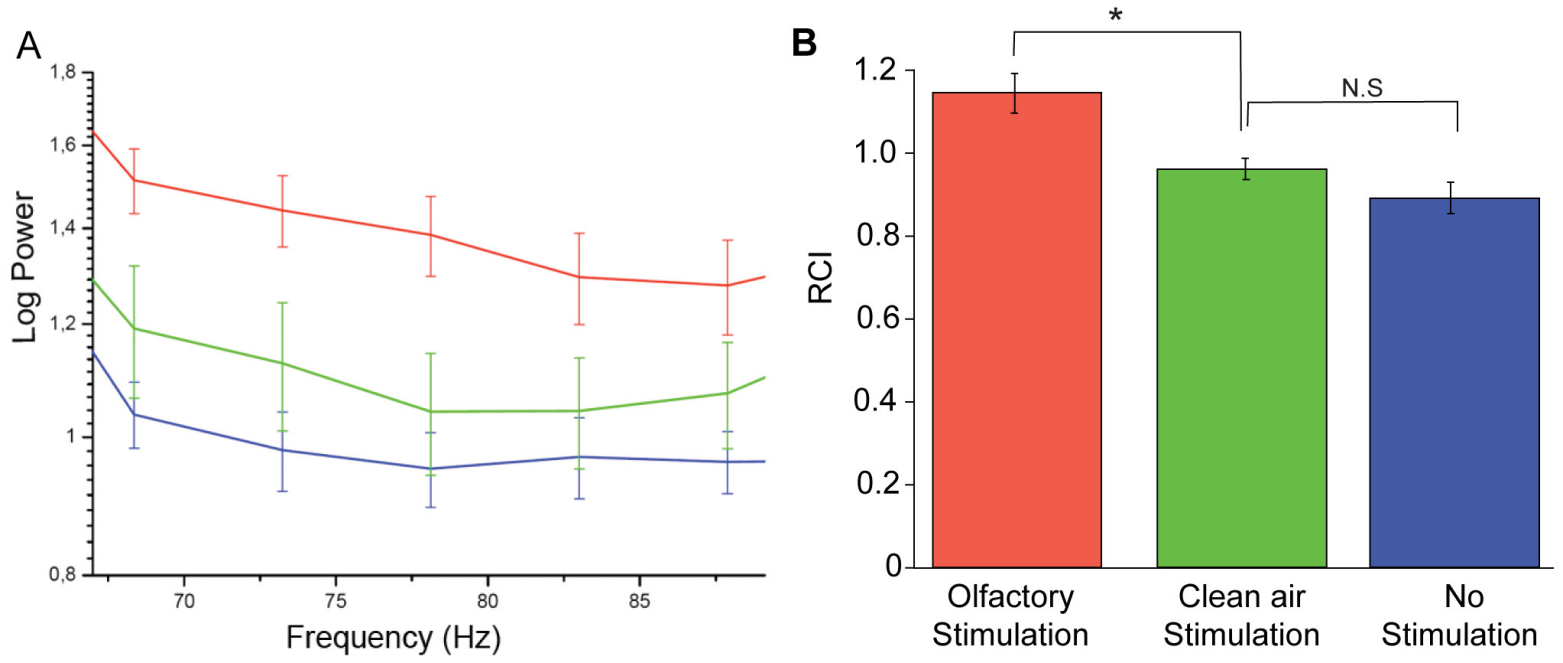
Response to odours. (A) Average (±SEM) of the 70–80 Hz power during baseline (blue), odour (red) and air (green) stimulation in a sample fly. Each line is an average of eleven recordings performed randomly on the same fly. (B) Average 70–80 Hz RCI (±SEM) at baseline (blue) and during olfactory (red) and air (green) stimulation. Olfactory stimulation showed a significant increase in the 70–80 Hz RCI compared to baseline and air stimulation. n = 3 flies, 11 recordings of each condition per fly; Significance was first assessed with an ANOVA *p* = 0.011. Afterwards a post-hoc two-tailed t-test was performed in between the different conditions; a single asterisk (*) indicates *p*<0.05, and *N.S* indicates *p*>0.05.

We first tested that the increased 70–80 Hz power is a neural correlate of olfactory processing and not an electrical artifact or a response to mechanical stimulation produced by airflow. We stimulated flies in a random sequence of odour and air puffs separated by times without stimulation. As the absolute power of signals recorded varied considerably across flies, we calculated an index measuring the rate of change in the 70–80 Hz power (Rate of change index: RCI; see Methods), which we will refer to as 70–80 Hz response. This is a normalized ratiometric index analogous to that used to describe front-to-back differences in visual salience responses [18], that uses a normalization by the mean responsiveness of each fly. We found a significant increase of the 70–80 Hz response during the odour stimulation compared to a puff of clean air or to the power during the no stimulation periods. (**Fig. 2**; *p* = 0.037 and *p* = 0.019, for the air puff and resting conditions respectively, n = 3 flies, 11 recordings per condition per fly). This result confirms the specificity of the response to olfactory stimulation.

### Recurrent olfactory stimulation reduces the 70–80 Hz response to odours

**Figure 3:**
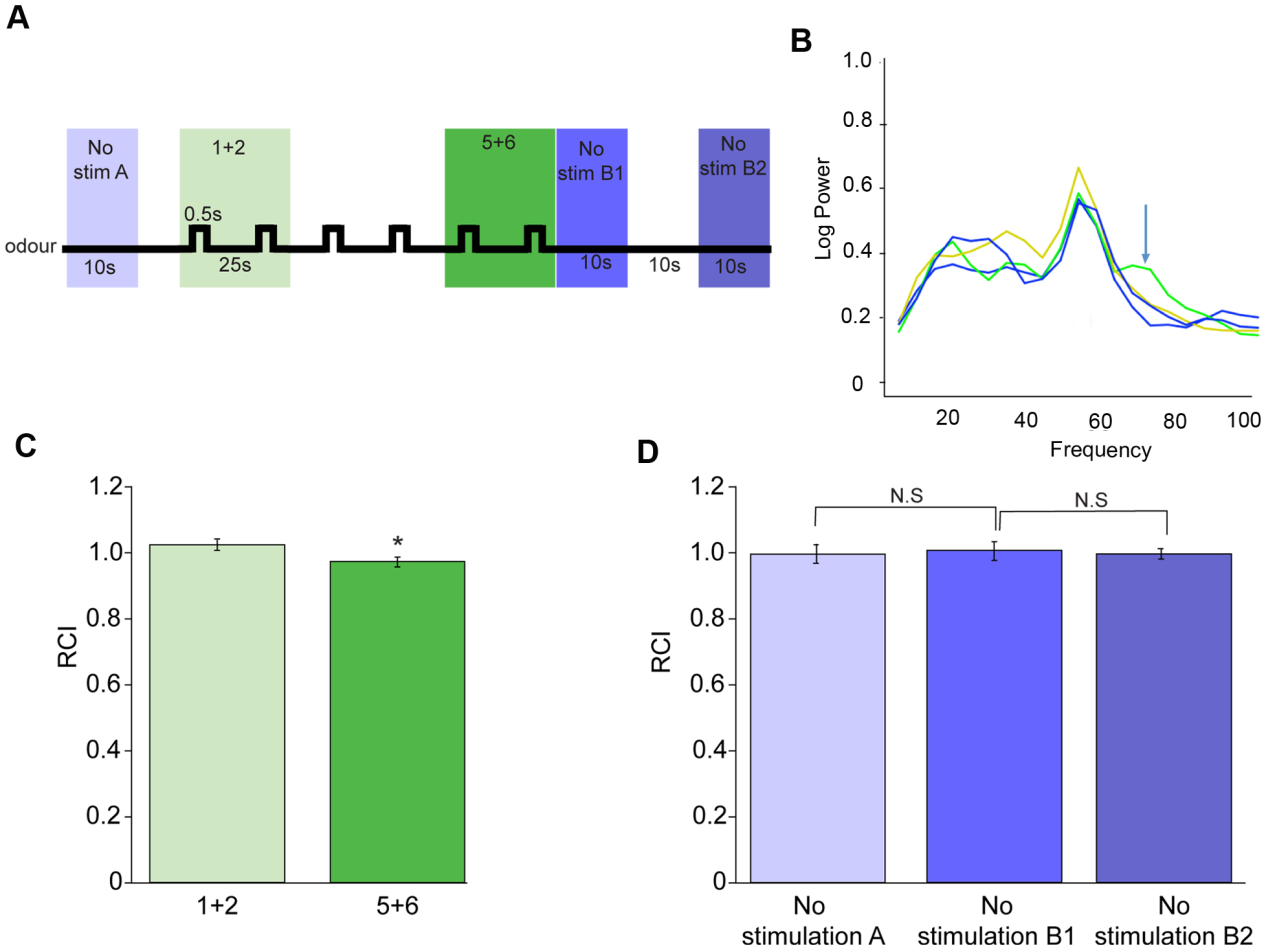
Recurrent olfactory stimulation reduces the 70–80 Hz response to odours. (A) Diagram illustrating the protocol. Flies were exposed to 6 consecutive 500 ms odour pulses spaced 25 s. (B) A sample power spectrum of brain frequencies (1–100 Hz) in response to the first odour presentation (green), compared to the sixth odour presentation (yellow), and unstimulated periods before and after the protocol (blue). The arrow points to the 70–80 Hz response to the first olfactory stimulation, and its specific reduction during the sixth exposure. (C) Average 70–80 Hz RCI (±SEM) during the presentation of the first two odour puffs (1+2, light green) compared to the response to the last two odour puffs (5+6, dark green). (D) The baseline response does not change during the protocol. Significance was assessed by two-tailed t-test; a single asterisk (*) indicates *p*<0.05, and *N.S* indicates *p*>0.05.

We then reasoned that if the 70–80 Hz response to odours is a trace of higher order olfactory processing, it should change upon exposure to olfactory protocols that might change the value of the olfactory stimuli being presented to the fly. To pursue this question, we first investigated the relation of the 70–80 Hz response to recurrent olfactory stimulation. We stimulated flies repeatedly using a modification of a protocol used before to induce behavioural habituation [19]. Our protocol consisted of 6 consecutive 500 ms odour presentations spaced 25 s apart (**Fig. 3A**). This protocol is known to produce neither receptor adaptation nor any changes in the response profile of projection neurons [20], [21]. The peak in responsiveness at 70–80 Hz during the first olfactory odour presentation (**Fig. 3B**, green line, arrow) was reduced during the sixth odour presentation (**Fig. 3B**, yellow line), without affecting the 70–80 Hz power at rest (**Fig. 3B**, blue lines). **Fig. 3C** shows averaged 70–80 Hz responses to the first 2 odour presentations, compared to the significantly decreased signal to the last 2 odour presentations of the series(**Fig. 3C**. *p* = 0.025; n = 7 flies). This protocol does not affect the RCI at rest (**Fig. 3D**). Thus, the fly's 70–80 Hz response decreases after repeated stimulus presentations.

### Electric shock stimulation increases the 70–80 Hz response to odours

**Figure 4:**
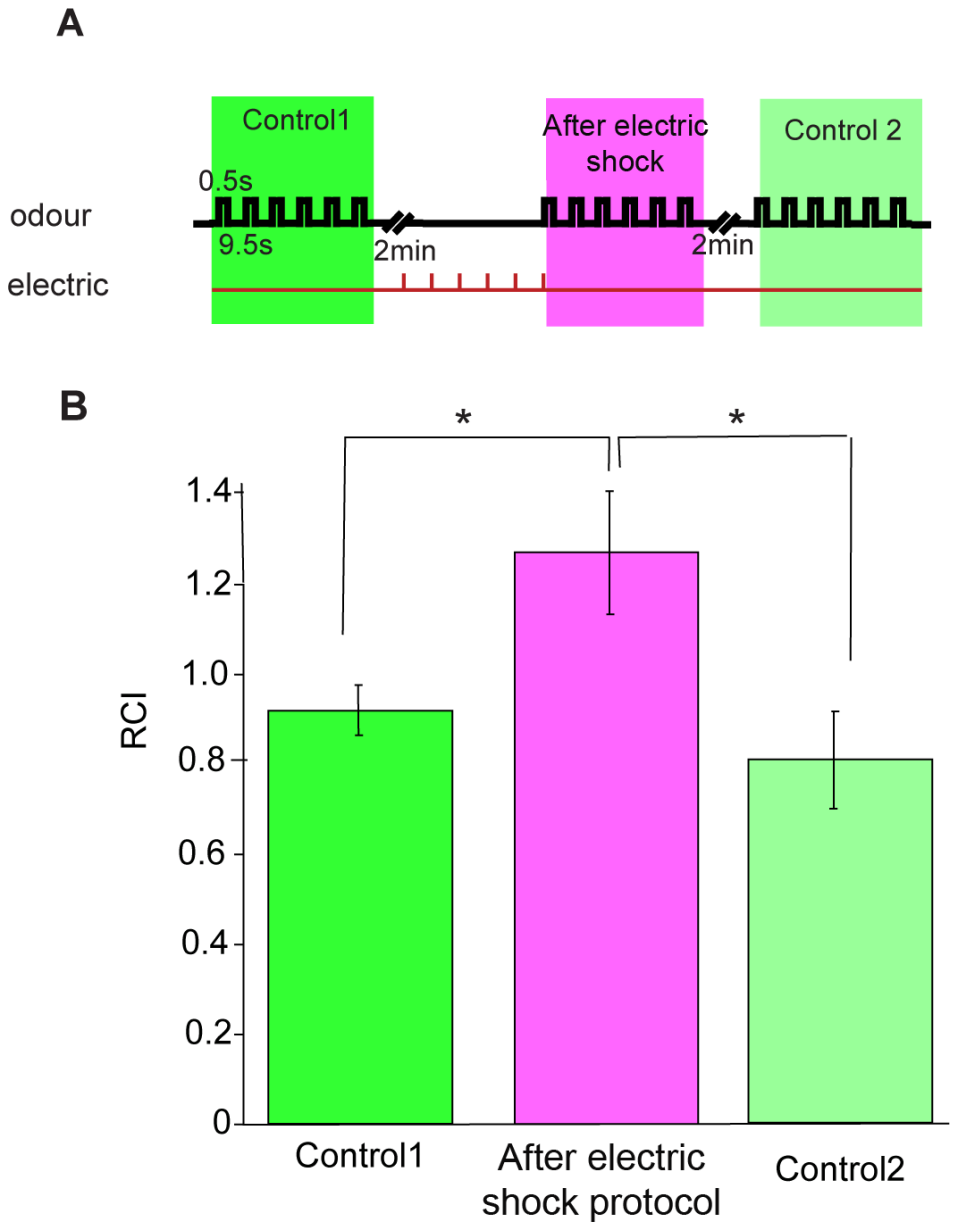
Electric shock stimulation increases the 70–80 Hz response to odours. (A) Diagram illustrating the protocol: 6 pulses of electric shock spaced 10 s. (B) Average 70–80 Hz RCI (±SEM) to 6 pulses of odour spaced by 10 s after the electric shock protocol (pink), compared to a control before the protocol (bright green), and to post-protocol control after 2 minutes of rest (light green). Significance was first assessed with a Kruskall Wallis test *p* = 0.027. Afterwards a post-hoc Wilcoxon test was performed in between the different conditions; a single asterisk (*) indicates *p*<0.05, and *N.S* indicates *p*>0.05.

We next wondered whether the 70–80 Hz response could be modulated in the opposite direction. We reasoned that if the signal in response to odours decreases after a protocol of repeated stimulation, then it might be possible to observe an increase in the response to odours after stimulating flies with a protocol that presumably increments their alert, such as a series of electric shocks. Our protocol consisted on a series of 6 mild electric shocks 10 seconds apart from each other. We found that the 70–80 Hz RCI to 6 consecutive odour puffs spaced 10 seconds apart from each other was significantly higher after the flies had been exposed to the electric shock protocol, when compared to the response of the flies to the same stimuli applied before the electric shocks (**Fig. 4B**. *p* = 0.011; n = 7 flies). The response to the olfactory stimulus returned to baseline levels after 2 minutes of rest (**Fig. 4B**. *p* = 0.037; n = 7 flies). As expected, the 70–80 Hz response to odours increases after a sensitization-like protocol, in contrast to the decrease after the habituation-like protocol.

## Discussion

We have recorded local field potential oscillations from the central brain of *Drosophila* in response to olfactory stimulation. The 70–80 Hz signal that we have recorded during olfactory stimulation can be modulated in opposite directions by two different protocols. We speculate that these protocols might change the adaptive value of the odour to the fly.

The signal we recorded increases when an odour is presented for the first time but it decreases after repeated stimulations, so it may constitute a neural correlate of olfactory novelty. We suggest that this effect emerges from higher order central processing in the protocerebrum. This is supported by previous studies in which the same protocol has been shown to produce no changes in the antennal lobe output neurons; notably a very similar protocol has been show to produce behavioural adaptation [19]–[21].

The 70–80 Hz response to olfactory stimulation increases after a series of electric shocks have been delivered to the fly. Classically, sensitization is defined as a non-specific increase in a behavioural response after the presentation of a sensitizing stimulus [22]. In bees it has been shown that the application of sucrose to the proboscis as sensitizing stimulus enhances proboscis extension response to an odour by sensitization of the reflex [23]. Sensitization of odour responses can also occur by using a noxious sensitizing stimulus instead of an appetitive one [24]. Asztalos et al. [24] showed in *Drosophila* that the olfactory jump response to benzaldehyde can be sensitized using a mechanical stimulus. To our knowledge, there is no detailed study on olfactory sensitization using an electric shock as sensitizing stimulus. Most studies incorporating electric shock stimuli used sensitization as a control for associative learning behaviour [25]–[27], but none of them aimed to study the effects of sensitization *per se*. Although our sensitizing stimulus was electrical and not mechanical, the increased signal in response to odours we observe in the 70–80 Hz range after our electric shock protocol might be interpreted as a neural correlate of olfactory sensitization, although definitive behavioural proof is still missing.

Although our results are based on the use of a single odour, benzaldehyde, it is tempting to speculate that the LFP changes we observed in the 70–80 Hz range are a general feature of higher order olfactory processing. A recent report has shown that unlike other odourants (3-octanol, n-amyl acetate, and 4-methyciclohexanol), benzaldehyde does not induce intensity-specific memories, although intensity does affect the establishment and recall of benzaldehyde memory [28]. These results do not constitute a caveat for our experiments, as the concentration of benzahdehyde was constant across our experiments. Benzaldehyde has been used to induce behavioural habituation and sensitization [19], [24], as well as associative memory in *Drosophila* [29], [30]. Furthermore, a previous study that reported the 70–80 Hz increase in LFP during olfactory stimulation used a complete different odour (banana extract) [18], which supports the generality of our findings.

The signals recorded here present basic features of a neural correlate of olfactory salience, similar to the 20–30Hz signal found by van Swinderen and Greenspan [18] to be a physiological signature of visual salience. However, further experiments will be needed to clarify the exact nature and significance of the signals recorded here.

The fruit fly's LFP responses share several key features with physiological correlates in the 40–60 Hz range of visual selective attention in monkeys and humans [31]. For example, amplitude increases with salience, which can be increased by either an unconditioned stimuli or by novelty. [32]. Furthermore, a recent study in rats reported that, during difficult odour discrimination tasks, strong synchronous oscillations appeared in the olfactory bulb field potential. These oscillations were not present when the task was easy and could reflect an increased attention [33]. Therefore in flies, as it has been suggested before in mammals [31], neural synchronization may be a common neural mechanism involved in complex cognitive tasks like arousal, perceptual integration, and attentional selection. Our findings in the olfactory system, together with the findings of others in visual attention [18], indicate that despite the fruit fly's lack of neuroanatomical homology with primates, *Drosophila* might have analogous mechanisms of establishing salience and directing selective attention to its environment.

## Materials and Methods

### Fly cultures and crosses

Flies were cultured at 25°C on brewer's yeast, dark corn syrup, and agar food. Only flies younger than 4 days were used in physiological experiments. The genotype of the flies used was yw;+;UAS GFPnls-Gal4 C739. C739 targets Gal4 expression to the lobes of the mushroom bodies.

### Set-up

Flies were first introduced, under a dissection scope, in a plastic pipette tip with the tip modified to allow only the head of the flies to pass through. Once the flies were positioned with the head outside the tip, they were immobilized applying low temperature melting wax in the proboscis and thorax. Small incisions were made in the right eye and in the central ocelli in order to facilitate the entrance of the electrodes. The fly was then positioned under the florescence microscope with a 20× air objective.

### Electrophysiology

Recording electrodes were placed in the medial protocerebrum between the mushroom bodies. Briefly, the electrode was positioned on top of the central ocelli at an angle of approximately 45 degrees with respect to the fly's main axis, and from there it was inserted 50–75 µm through the central ocelli until it could be visualized between the mushroom bodies (visible in green under UV light). Ground electrode was placed inside the right eye through a small incision made under the dissection microscope. Before recordings, a light responsiveness test was performed using a small flashlight. Only flies that responded to light stimulation were used for further experiments. After this test, the rest of experiments were performed in darkness. Recording and ground electrodes were 10 µm tip tungsten electrodes (*Frederick Haer and Co.*, *12mm plus pin*). Resistance between the brain and eye electrodes was 0.2–0.8 MΩ. Signals were fed to an amplifier (*Brownlee Precision*, *model440*) in which signals were amplified 10,000 times and band pass filtered between 1 and 150 Hz. Amplifier output was digitized (*LPBF-01G*, *npi instruments*) and then stored at a sampling rate of 10 KHz with custom made software [34].

### Olfactory stimulation

Olfactory stimuli were delivered through a glass vial fitted with a silicone stopper on which the odour compound (benzaldehyde diluted 100-fold v/v in water) was deposited. Airflow speed was set to 0.8 L/min. The odour stimulus was pulsed by means of a solenoid-activated valve controlled by an electronic stimulator (*Bio-electronic shop*, *Caltech. Pasadena*, *California*, *USA*). It was delivered to the fly using a glass tube ending in a 3 mm diameter and positioned 10 mm from the fly antennae and orthogonal to them. For the initial experiments to rule out mechanical or electrical artifacts, recordings lasted 1 s and were spaced randomly between 1 and 3 minutes. During the 1 s periods we randomly administrated either a puff of air (airflow was delivered directly to the fly without passing through the vial containing the odour and passing instead through an empty vial), or a puff of odour, or we recorded 1 s of activity in the absence of any stimuli. For recurrent olfactory stimulation experiments, stimulus duration was 500 ms consisting of six pulses separated by an interval of 25 s. For sensitization experiments, olfactory stimulus duration was 500 ms and each pulse was separated by 9.5 s.

### Electric stimulation

The pipette tip in which the flies were introduced was perforated at the level of the fly thorax. A conductive gel (*Ten20 Conductive*, *EEG paste*, *D.O Weaver and Co.*) was introduced in the holes to make contact between the thorax and the shock delivery electrodes connected to a computer controlled stimulator (*ISO-STIM01M npi instruments*). Shock delivery electrodes were placed in contact with the conductive gel on the thorax of the fly during the stimulation period and quickly moved away during the recording period. The electric shock pulses were 90 V, 100 ms long steps spaced 9.9 s from each other, making a total of 6 shocks over a minute for the sensitization protocol.

### Data analysis

Recordings were acquired and analyzed with custom made software written in Matlab language (*MathWorks*, *Natick*, *MA*). Every recording was fragmented in bins of the relevant size and the 70–80 Hz power calculated for each bin. The bin size was 1 second for the experiments in figure 1 and 2, 10 seconds for figure 3, and 1 minute for the experiments in figure 4. Ratio of change index (RCI) was calculated as *I* = *A*/*X*, being *A* the value of the power at 70–80 Hz in the bin that is being analyzed, and *X* the average of the value of the power at 70–80 Hz for all the bins that are being compared in a plot. For example, in figure 4:
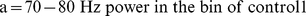


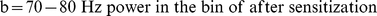


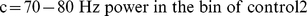


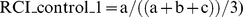


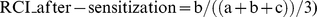


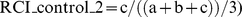
Normally distributed data were analysed using two-tailed t-test or one-way ANOVA analysis for comparison of multiple datasets. For non-normally distributed data the Wilcoxon rank-sum test was used or Kruskal Wallis test for comparisons of multiple datasets.
